# Anti-C1q Antibodies as Occurring in Systemic Lupus Erythematosus Could Be Induced by an Epstein-Barr Virus-Derived Antigenic Site

**DOI:** 10.3389/fimmu.2019.02619

**Published:** 2019-11-07

**Authors:** Kinga Csorba, Lucia A. Schirmbeck, Eylul Tuncer, Camillo Ribi, Pascale Roux-Lombard, Carlo Chizzolini, Uyen Huynh-Do, Dominique Vanhecke, Marten Trendelenburg

**Affiliations:** ^1^Clinical Immunology, Department of Biomedicine and Division of Internal Medicine, University and University Hospital Basel, Basel, Switzerland; ^2^Immunology and Allergy, Department of Internal Medicine, University Hospital Lausanne, Lausanne, Switzerland; ^3^Division of Immunology and Allergy, Department of Medicine, University Hospital and University of Geneva, Geneva, Switzerland; ^4^Division of Nephrology and Hypertension, University Hospital Bern, Bern, Switzerland

**Keywords:** systemic lupus erythematosus, anti-C1q antibody, A08 epitope, Epstein-Barr virus, EBNA-1, C1q deficient mice, autoimmunity, molecular mimicry

## Abstract

Previous infection with Epstein-Barr virus (EBV) is believed to trigger autoimmunity and to drive autoantibody generation as occurring in patients with systemic lupus erythematosus (SLE). Complement C1q and autoantibodies targeting it (anti-C1q) are also considered to be involved in the pathogenesis of SLE, independently of the impact of environmental insults. Still, the circumstances under which these autoantibodies arise remain elusive. By studying a major antigenic site of C1q targeted by anti-C1q (A08), we aimed to determine environmental factors and possible mechanisms leading to the development of anti-C1q. First, we determined antigenic residues of A08 that were critical for the binding of anti-C1q; importantly, we found the binding to depend on amino-acid-identity. Anti-C1q of SLE patients targeting these critical antigenic residues specifically cross-reacted with the EBV-related EBNA-1 (Epstein-Barr virus nuclear antigen 1)-derived peptide EBNA348. In a cohort of 180 SLE patients we confirmed that patients that were seropositive for EBV and recognized the EBNA348 peptide had increased levels of anti-A08 and anti-C1q, respectively. The correlation of anti-EBNA348 with anti-A08 levels was stronger in SLE patients than in matched healthy controls. Finally, EBNA348 peptide-immunization of C1q^−/−^ mice induced the generation of cross-reactive antibodies which recognized both the A08 epitope of C1q and intact C1q. These findings suggest that anti-C1q in SLE patients could be induced by an EBV-derived epitope through molecular mimicry, thereby further supporting the pathogenic role of EBV in the development of SLE. Considering the role of C1q and anti-C1q, modifying the anti-EBV response might be a promising strategy to improve the course of the disease.

## Introduction

Systemic Lupus Erythematosus (SLE) is an autoimmune disease characterized by the occurrence of autoantibodies, resulting in a broad spectrum of immunological and clinical manifestations. Among the severe manifestations of SLE, renal involvement is the most frequent and, in its most aggressive forms, can lead to renal failure.

Besides the heterogeneous clinical presentation, the pathogenesis of SLE is most likely due to a combination of genetic predisposition with hormonal and environmental factors, such as ultraviolet light, chemicals, medication, and parasitic, bacterial or viral infections. The hypothesis that viral infections might initiate autoimmunity is supported by clear epidemiological, clinical and experimental data. Viruses such as Cytomegalovirus, Parvovirus B19 and Epstein-Barr virus (EBV) have been well described to be environmental triggers of SLE (1–4). EBV infection attracted specific attention because SLE patients seem to form aberrant antibodies, with the potential to cross-react with autoantigens by means of molecular mimicry. Moreover, experimental immunization of animals with EBV-derived peptides has been shown to induce typical SLE autoantibodies (5–8).

In humans, the characteristic autoantibodies can appear many years before the first clinical manifestation of the disease (5, 9). Among these autoantibodies, some have been considered to be pathogenic based on their close association with disease activity and their target being involved in the pathogenesis of SLE. In this sense, autoantibodies against C1q (anti-C1q), the starter molecule of the classical pathway of complement, are of specific interest. On the one hand, anti-C1q correlate with overall disease activity (10) and the occurrence of severe lupus nephritis (11–13), making them an important diagnostic marker. On the other hand, C1q is deposited in inflamed tissues and associated with complement activation as well as the deposition of immune complexes (14). Thus, anti-C1q have the potential to accelerate the course of disease (15, 16). One important rationale for the role of C1q and anti-C1q in SLE is based on the so-called “waste disposal” hypothesis. This assumes that SLE is driven by a defective clearance of dying cells that can become antigenic and drive autoimmunity. In fact, C1q has been described to accelerate the clearance of self-antigens generated during apoptosis, and macrophages from C1q-deficient mice and humans show a defective clearance of apoptotic cells *in vitro* (17, 18). In SLE patients, this physiological function of C1q is likely to be altered by the binding of anti-C1q. Anti-C1q most frequently target a cryptic epitope on the collagen-like region of C1q. In a previous study, we identified a major linear epitope of C1q located on the collagen-like stalk region of C1q, the so-called A08. Autoantibodies against this single peptide epitope (anti-A08 IgG) have also been found to correlate with SLE disease activity and lupus nephritis (19, 20).

The aim of our study was to characterize the antigenic site A08 of C1q with the goal to determine a possible connection to environmental antigens, which could trigger the development of anti-C1q. Revealing mechanisms leading to the generation of anti-C1q as a consequence of exposure to specific pathogens would allow specific and preventive immunotherapeutic approaches with the aim to reduce the incidence and severity of systemic autoimmunity.

## Materials and Methods

### Serum/Clinical Serum Samples

Sera/plasma from patients included in the Swiss Systemic Lupus Erythematosus Cohort Study (SSCS) were selected based on the availability of biomaterial and complete SLE disease activity measures at the time of inclusion and sampling. SLE patients (*n* = 180) fulfilled at least 4/11 classification criteria of the American College of Rheumatology (ACR). On average, patients had a median age of 43 years (range 16–84, 86.2% females), disease duration of 10.5 years (since diagnosis) and a median SLEDAI score of 4 at the time-point of blood sampling (see Supplementary Table 1). Additionally, sera from selected SLE patients (*n* = 17) (introduced in a previous study (19) and with known anti-C1q and anti-A08 IgG levels covering a broad range of titers) were also included in the study (Supplementary Table 2). Normal human sera (NHS) from 189 age- and sex-matched blood donors (median age 49 years, range 19–81, 85.6% females) recruited during routine donations at the Blood Transfusion Center Basel (Blood Transfusion Center Basel, Swiss Red Cross, Basel, Switzerland) were used as controls. All samples were anonymized.

We defined SLE patients with a Physician's Global Assessment (PGA) of 1 or higher as having active disease. Additionally, active patients included in Supplementary Table 1 and in the correlation analysis were also VCA and EBNA-1 positive.

SSCS was approved by the Ethical Committee of the Canton Basel, Switzerland (Ref No EK 262/06) and fulfilled the guidelines of the most recent Declaration of Helsinki. Patients gave written informed consent for the study participation.

### Mice

Six to nine week-old C57BL/6N and C1qa^−/−^ female mice (21) with a body weight of approximately 19 g, were obtained from the animal facility of the Department of Biomedicine, where they were maintained under pathogen-free conditions. The C1qa^−/−^ mice were on a C57BL/6N genetic background backcrossed for at least 10 generations. Mice were distributed randomly to the different groups. Immunizations were performed (s.c.) on mice narcotized with isoflurane. This study was carried out in accordance with the recommendations of the Swiss welfare legislation (consisting of Animal Welfare Ordinance, Animal Welfare Act and the Animal Experimentation Ordinance) The protocol was approved by the Cantonal Commission for Animal Experiments, and the Federal Food Safety and Veterinary Office (2633/23801), and performed by authorized staff.

### Peptides

**Figure 1:**
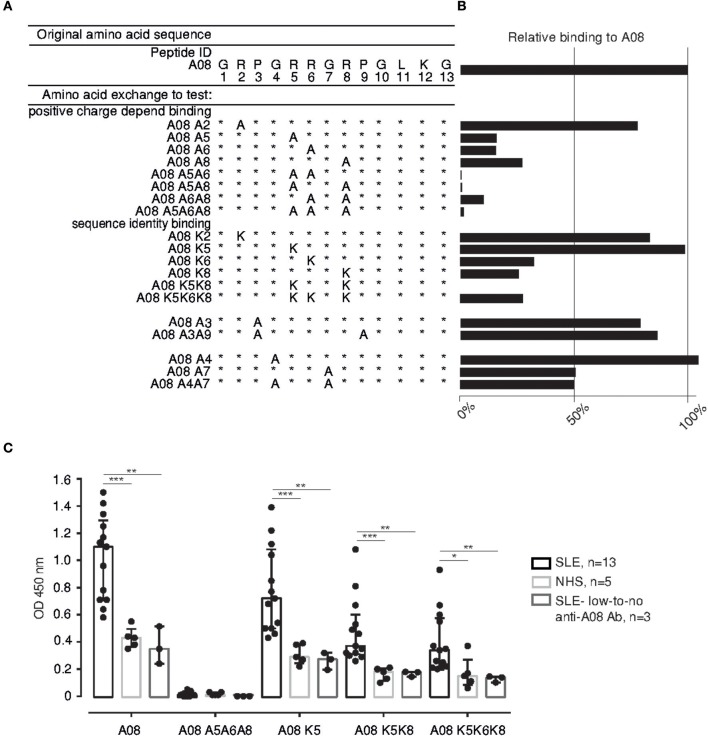
Identifying the binding core in the C1q-derived epitope A08. **(A)** Variations of the A08 peptide with 1, 2, or 3 AAs substitutions: Arg (R) was exchanged for Ala (A) and Lys (K) in order to alter the net charge of the peptide (at neutral pH) or to modify its sequence. **(B)** Serum from the reference SLE patient shows a specific binding pattern to the A08 peptide variations. Binding is expressed as percentage relative to the 100% binding to the original A08. **(C)** Serum from SLE patients with anti-A08 IgG (*n* = 13), with low-to no levels of anti-A08 IgG (*n* = 3) and from healthy donors (NHS) (*n* = 5) were tested on a selection of A08 peptide variations. Bar graphs show median with IQR. *p ≤ 0.05, **p < 0.01, ***p < 0.001, ****p < 0.0001. Ab, antibody.

N-terminally biotinylated and non-biotinylated peptides with >95% purity were synthesized by GenScript (USA) and peptides & elephants GmbH (Germany). The A08 peptide (sequence): GRPGRRGRPGLKG is derived from the A chain of C1q. A08 peptide variants used for the AAs (amino acids) exchange experiments are summarized in Figure 1A. The AAs sequence of microorganisms-derived peptides and peptides derived from the human proteome having a sequence identity to the core of A08, as well as a scrambled version of the A08 peptide are summarized in Supplementary Table 3.

### Sequence Alignment

The vertebrate C1q sequences were extracted from the vertebrate reference sequence database of NCBI and a semi global MALT alignment was performed with a 25 AAs long query sequence containing the A08 sequence. Significance of alignment was judged by the *e* value. The species selected from the generated file are summarized in Supplementary Table 4.

Protein BLAST with the GRRGR core sequence was carried out on the human proteome (*Homo sapiens*, taxid 9606) of the UniProtKB/Swissprot sequence database. For this we looked at the first 50 hits (all had a score of 18.5, with an *e* value of 17) and selected proteins based on the following keywords listed in the protein database of NCBI: secreted, cell death-related, immunity-related, collagen. Microorganisms-related proteins containing the core sequence were retrieved in a similar way. The organisms' dataset was narrowed down to certain pathogens based on their association with the occurrence of SLE and/or assumed causal relationship (1, 22). A list of selected peptide sequences is summarized in Supplementary Table 3.

### ELISA for Assessment of Antibodies in Human and Mouse Serum

#### EBV Seroprevalence and Anti-C1q in Clinical Samples

The seroprevalence of EBV was determined by serum ELISAs for anti-VCA IgG (Meridian Bioscience Inc.) and anti-EBNA-1 (Epstein-Barr virus nuclear antigen 1) IgG (Abnova). We determined the anti-VCA levels in a semi-quantitative way, using kit calibrators that allow the unit value determination of each sample. The serum was diluted 20-times and the secondary detection antibody was a goat-anti-human IgG-HRP (γ-chain specific). For the anti-EBNA-1 assay the recombinant immunodominant epitopes of the EBNA-1 were incubated with 100-times diluted serum samples. The antigen-antibody complexes were then recognized by a horseradish peroxidase labeled anti-human IgG-HRP (γ-chain specific) and the enzymatic reaction was visualized with TMB as a chromogenic substrate. Both commercially available kits provide standardized solutions to be included on each assay plate to calculate an individual standard curve. This standard curve covers the linear part of the curve and includes a negative control. The AUs were directly derived from the standard curve without modifying calculations. The assay for anti-EBNA-1 included the following controls: negative, 20, 45, 90, and 500 AU. The manufacturers' protocol gives 12 AU as cut off for positivity. The assay for anti-VCA included the following controls: negative, 12, 396, 725 AU and positive. The manufacturers' protocol gives 150 AU as cut off for positivity. When analyzing the associations between anti-EBNA-1 IgG and anti-EBNA348 IgG, it is important to note that the anti-EBNA-1 IgG kit might not contain the sequence of the EBNA348 peptide.

Anti-C1q IgG levels were determined using a commercially available assay (Bühlmann Laboratories, Schönenbuch, Switzerland) following the manufacturer's instructions. Standardization of the anti-C1q ELISA is based on defined calibrators which are calibrated against an internal reference sample. The reference sample is derived from a plasmapheresis of a highly positive and clinically well-defined patient. The cut-off is set at 15 AU by the manufacturer.

As control autoantibodies, anti-ß2GPI levels were measured according to a published protocol (23).

All ELISAs were performed in one step with each sample in duplicates.

#### Peptide ELISA for Clinical Serum Samples

Polystyrene plates (MaxiSorp, Nunc) were coated with 350 ng/well NeutrAvidine (Thermo Fisher) in 0.2 M bicarbonate buffer (pH 9.6) for 1 h at 37°C. A 2 h incubation with 500 ng/well-biotinylated peptides followed. Sera [diluted 1: 500 in 0.05% Tween20, 0.4 M NaCl-PBS (w/v)] were added to the plate for 1 h.

Bound antibodies were detected by a 10,000-fold diluted, alkaline-phosphatase (AP) labeled goat anti-human IgG-Fc gamma chain specific secondary (#109-055-008 Jackson Immuno Research). OD was read (405 nm) and analyzed using a Synergy H1 Hybrid ELISA reader and Gen5 Analysis Program (BioTek). All incubation steps were performed at 27°C if not stated differently, and were followed by a washing step using 0.05% Tween20-PBS (w/v). For data analysis results were normalized: all measured values were expressed in units relative to the OD values of the reference SLE serum. The reference serum had high level anti-EBNA348 IgG and high level anti-A08 IgG. The corresponding OD values on each plate were set to 100 AU. The reference serum was included on each plate/in each experiment. Samples were tested in duplicates. A conservative cut-off, considering the non-normal distribution of the values, was defined by the median value (AU) plus 3 median absolute deviations of the EBV seropositive normal human donors: 105 AU for the anti-EBNA348 IgG- and 82 AU for the anti-A08 IgG assay.

The peptide ELISAs with human serum were done twice with each sample in duplicates. The results shown are originating from one experiment though with the other one confirming the results.

#### Peptide, Anti-C1q and ANA ELISA for Mouse Sera

The presence of anti-peptide and anti- human C1q IgG in mouse serum was confirmed by an assay similar to the one described above. Polystyrene plates (MaxiSorp, Nunc) were coated with 500 ng/well NeutrAvidine or 350 ng/well-purified human C1q (Complement Technology) protein, respectively, in 0.2 M bicarbonate buffer (pH 9.6), o/n at 4°C. A 2 h incubation with 500 ng/well-biotinylated peptides followed on the NeutrAvidine coated plates. The mouse sera (diluted 1:500 in 0.05% Tween20-PBS (w/v) or 1:10 in 0.5 M NaCl in 0.05% Tween20-PBS) were incubated for 1.5 and 1 h, for the peptide and the protein assays, respectively. Point five molar NaCl-Tween20-PBS (= high-salt) buffer was used to prevent immune complex binding to intact C1q. Bound IgG was detected by a 20,000-fold diluted horseradish peroxidase (HRP)-conjugated Fcγ-specific rabbit anti-mouse secondary (#SAB3701037, Sigma) and TMB substrate (BD Bioscience). For the protein assay the secondary too was diluted in salt-containing buffer. OD was read (450 nm) and analyzed as described above. Incubation steps, if not otherwise mentioned, were at room temperature, and were followed by washing with 0.05% Tween20-PBS. For data analysis results were normalized. The standard positive sample (from an MRL-lpr mouse with high anti-EBNA348-, anti-A08-, and anti-C1q IgG levels) was included in each experiment/plate. Additionally, for the peptide ELISAs, the signal obtained from incubating sera (human or murine) with NeutrAvidine was considered background, and was subtracted from the specific signal. Samples were tested in duplicate. The cut-off for the mouse anti-human C1q ELISA was set at 0.061 OD by calculating the median OD value at 450 nm + 3 median absolute deviations of the OD values of adjuvant-injected control mice. Anti-C1q ELISAs were repeated twice, each time with samples in duplicates; data shown are from a single experiment. For the data shown, anti-A08 and anti-EBNA348 assays were performed once, with samples in duplicates. Anti-C1q measurements were performed twice, with duplicates for each sample; data included originated from one experiment.

Serum anti-nuclear antibodies (ANA) were determined by a commercially available mouse ANA ELISA Kit (Alpha Diagnostic, San Antonio, TX), according to the manufacturer's protocol. Plates precoated with purified nuclear antigens were incubated with mouse sera [1:500 in 0.05% Tween20-PBS (w/v)] for 1 h. Bound antibodies were detected with the anti-mouse IgG + IgA + IgM (H + L) HRP conjugate and the enzymatic reaction was visualized by adding TMB substrate. The reaction was stopped with 0.25 M sulphuric acid and the absorbance measured at 450 nm using a micro plate reader (Bio-Tek). Test sample concentrations were interpolated using a four-parameter logistic calibration curve to the standards provided by the manufacturer (50, 200, 500, and 1,000 U/mL). The experiment was performed once, with duplicates for each sample.

#### Inhibition Assay

Inhibition of serum IgG binding to C1q was performed as described above, except that patient sera were diluted 300-times in 0.4 M NaCl-PBS buffer and pre-incubated with soluble EBNA-1 for 2.5 h at room temperature on a shaking platform (300 rpm/min). A protein amount was used to reach a molar excess of ~3-fold the average amount of IgG present in NHS (12 mg/ml IgG). Prior to adding the pre-incubated and diluted patient sera to the C1q coated plate we added NaCl crystals to each tube in order to obtain a 1 M NaCl-PBS (= high salt) buffer, which is essential to prevent non-specific immune complex binding to intact human C1q (24). The sera were not diluted further. After 1 h incubation at room temperature on a shaker, bound IgG was detected by a 5,000-times diluted alkaline phosphatase-(AP)-conjugated rabbit-anti-human IgG (Promega) in 0.5 M NaCl-0.05% Tween20-PBS buffer (1 h, shaking). Subsequently, color development with AP substrate (Sigma-Aldrich) and reading of OD 405 nm at defined timepoints (12 ± 2 min after initiating the enzymatic reaction).

For each patient serum at least two conditions were pre-incubated in each experiment: the one that contained EBNA-1 in excess (different amounts) and the one that had no EBNA-1, but only the 0.4 M NaCl-PBS buffer. Samples were run in duplicates. Individual experiments were run 2 or 3 times, data shown are pooled.

Prior to use, the commercially available EBNA-1 protein (Advanced Biotechnologies 10-523-001) was subjected to an ~50 h dialysis, at 4°C. This step was necessary because the protein was initially solubilized in 6 M urea (buffer which is incompatible with most of the assays). We gradually removed the urea while increasing the NaCl concentration (up to 0.4 M-NaCl in PBS), a condition that according to the manufacturer allows the protein to slowly re-fold. After dialysis we centrifuged the soluble EBNA-1-0.4 M NaCl-PBS solution to remove possibly precipitated protein: 40 min, 4°C, at 10.000 × g and used it immediately for downstream analysis.

### Immunization of Mice With the EBNA348 (EBNA-1-Derived EBNA-1_348_) Peptide

In order to induce an EBNA-1-derived peptide-specific autoimmune response C57BL/6N (*n* = 10/group) and C1qa^−/−^ mice (*n* = 13/group) were immunized s.c. at the tail base with 100 μg of EBNA348 peptide: GSGGRRGRGRERARGGS emulsified in CFA (Invivogen). Booster injections followed after 2, 4, and 8 weeks with peptide in IFA (Invivogen). Mice of the control group (*n* = 5 C57BL/6N, *n* = 13 C1qa^−/−^) were immunized with PBS emulsified in CFA and IFA, respectively. CFA immunized mice were monitored closely and the general health condition of the animals was evaluated thrice a week throughout the experiment. Mice were weighed and bled before immunization and boosters, and 10 days after the 2nd and 3rd boost, for a period of 12 weeks. Sera were tested for autoantibody titers and cross-reacting antibodies. One C1qa^−/−^ mouse (control group) was excluded early in the experiment due to its considerably lower weight, and a C57BL/6N mouse (peptide-immunized group) died during the experiment, thus results and figures are reporting 12 C1qa^−/−^ adjuvant control mice and 9 C57BL/6N peptide immunized mice, if not mentioned otherwise. Immunization experiments were repeated twice in each strain to confirm the results; data shown are from single experiments.

The rationale behind our choice of the C1qa^−/−^ mouse strain was that we wished to reconstitute the cross-reactivity in a system where we have no intrinsic target nor preformed antibodies against the target (A08/C1q) interfering with the results as observed in lupus-prone mice (25, 26).

### Statistics

Non-parametric testing between two or more groups was performed by two-tailed Mann–Whitney-U or Kruskal-Wallis test, correlations were estimated by Spearman's rho, and contingency tables were analyzed by Fisher's exact test using Prism 7 software (GraphPad Software, La Jolla California USA, www.graphpad.com). Correction for multiple testing was done with the Bonferroni method. Statistical significance was considered with *p* ≤ 0.05; ^*^p ≤ 0.05, ^**^p < 0.01, ^***^p < 0.001, ^****^p < 0.0001.

## Results

### Binding of Anti-C1q to the Core of A08 Is Amino Acid Identity-Dependent

Previously, we described a major linear epitope of complement C1q targeted by anti-C1q of SLE patients, the A08 peptide. Antibodies binding to it were validated as a biomarker for active lupus nephritis (19, 20). We now characterized the A08 epitope and identified, by interspecies sequence alignment, the arginine in position 6 (Arg6) as unique to human C1q (Supplementary Table 4). We also tested whether the Arg and other amino acids (AAs) are essential for anti-C1q binding, by generating A08 versions (Figure 1A) with modified net charges [Arg to alanine (Ala)] or/and sequence [Arg to Lysine (Lys), glycine (Gly) to Ala]. Initially, we found that the Arg5, 6, 8, and the Gly7 are required for full anti-C1q binding (Figure 1B) thus forming a critical residue motif for antigenicity: RRGR. The extended screening, with higher numbers of sera from SLE patients (Supplementary Table 2 and Figure 1C) and normal donors confirmed the importance of the central contiguous RRGR AAs for anti-C1q binding. Regardless of single Arg residues being replaced by Ala or Lys, the signal, expressed as binding percentage relative to A08, dropped by 70–85%, except for A08K5 (Figure 1B). Furthermore, double and/or triple substitutions of the Arg led to progressive loss of antibody binding (Figures 1B,C). Additionally, exchanging arginines for neutral residues (Ala) abrogated the binding signal. However, the reconstruction of the peptide with a similarly charged AA (Lys), did not recover the signal (Figure 1C). Further on, sera of normal donors and of SLE patients with low-to no anti-A08 IgG recognized A08 and its variations to the same extent, but significantly less than the SLE patients with anti-A08 IgG (Figure 1C).

### Anti-C1q Directed Against the A08 Core Sequence Recognize the EBNA-1-Derived EBNA348 Peptide

**Figure 2:**
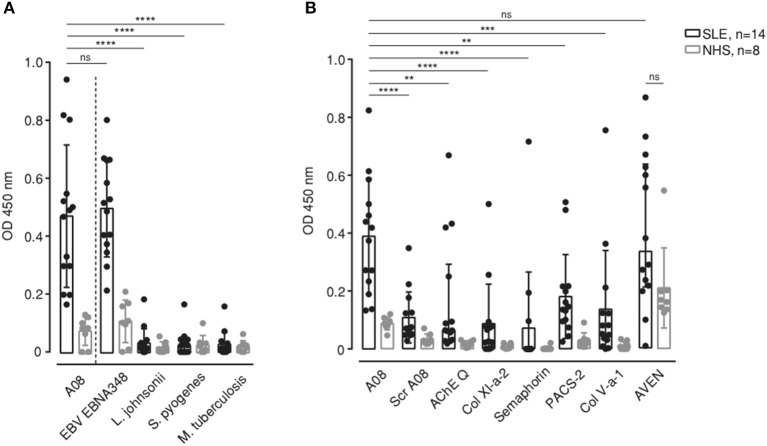
Binding to different microorganism-derived peptides **(A)** and human protein-derived peptides **(B)** containing the core AAs sequence of A08 was measured in SLE patients (*n* = 14) and healthy donors (NHS) (*n* = 8). Bar graphs show median with IQR. **p < 0.01, ***p < 0.001, ****p < 0.0001. ns, not significant.

While the full A08 peptide sequence is unique to human C1q, its core RRGR sequence occurs in other proteins too. Next, we aligned the A08 core sequence to the human proteome and to a microorganism-derived protein dataset and screened selected peptides (Supplementary Table 3). Regarding microorganism-derived peptides, it was striking that anti-C1q of SLE patients also recognized the EBNA-1-derived peptide “EBNA348,” i.e., a 17 AAs long peptide starting at position 348 of EBNA-1 (Figure 2A). The binding to A08 and EBNA348 was equally strong, while the level of binding to all other pathogen-derived peptides was significantly lower (*p* = 0.0001, for all differences in binding between A08 and the other pathogen-derived peptides), suggesting a specific binding of SLE sera to EBNA-1 of EBV. Additionally, we analyzed human protein-derived peptides for which we could not observe an overall, specific and strong signal, such as the one to EBNA348. Binding to A08 was significantly stronger when compared to other peptides (Bonferroni corrected significance: ScrA08 *p* = 0.0001, AChE Q *p* = 0.015, Col XI-a-2 *p* = 0.0001, Semaphorin *p* = 0.0001, PACS-2 *p* = 0.021, Col V-a-1 *p* = 0.0014), with the exception of cell death regulator Aven (AVEN). However, in this non-exclusive set of peptides only AVEN was recognized not only by SLE patients' but also by normal human donors' sera (Figure 2B), and this binding did not differ significantly (Bonferroni corrected *p* = 0.19), suggesting that the reaction to AVEN is not SLE-specific.

### EBV Seropositive SLE Patients Recognizing the EBNA-1-Derived EBNA348 Peptide Have Higher Levels of Anti-A08 IgG and Anti-C1q

As SLE patients might form an aberrant antibody reaction to the EBNA-1-derived peptide EBNA348, we next explored the association of antibodies against EBV with antibodies against intact C1q and the C1q-derived epitope A08. For this, we analyzed the overall seropositivity for EBV based on antibody titers against common EBV antigens in a large number of SLE patients being included in the Swiss SLE Cohort Study (SSCS).

**Figure 3:**
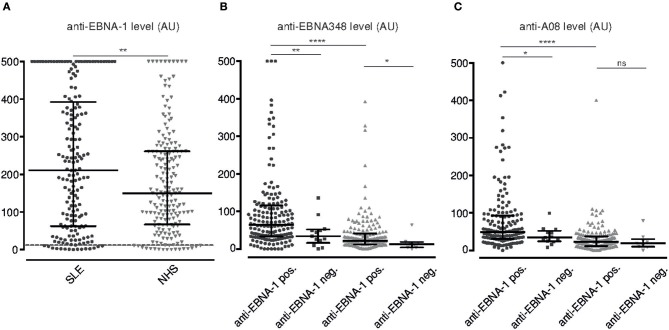
Serum levels of anti-EBNA-1 IgG, anti-EBNA348 IgG and anti-A08 IgG in SLE patients and normal human donors. **(A)** Anti-EBNA-1 IgG was measured in SLE patients (*n* = 180, •) and normal human controls (*n* = 189, 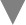). Dashed line indicates the kit-defined cut-off (12 AU). **p = 0.0084. Anti-EBNA348 IgG **(B)** and anti-A08 IgG **(C)** levels were analyzed in EBV seropositive sera further grouped based on their anti-EBNA-1 status. *N* = 165, •/ *n* = 13, ■, are anti-EBNA-1 positive and negative SLE patients, respectively. *N* = 171, 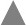 / *n* = 8, 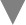, are anti-EBNA-1 positive and negative NHS, respectively. *p ≤ 0.05, **p = 0.0019 for **panel A** and **p = 0.0094 for **panel B**, ****p < 0.0001. Dot plots show median with IQR.

Overall EBV seropositivity, as defined by positivity for either anti-VCA IgG and/or anti-EBNA-1 IgG, was higher in SLE patients than in healthy controls (Table 1). Also, levels of anti-EBNA-1 IgG were higher in SLE patients (Figure 3A), which did not correlate with levels of total IgG (*r* = 0.1178).

**Table 1:** Statistical characteristics of EBV seroprevalence.

**Clinical test**	**SLE (*n* = 180)**	**NHS (*n* = 189)**	**OR (95% CI)**	***P*-value**
EBV seroprevalence	98.9%	94.7%	4.97 (1.07–23.02)	0.036
VCA seropositivity	98.3%	91.5%	5.45 (1.56–19.07)	0.0037
EBNA-1 seropositivity	91.7%	90.5%	1.16 (0.43–3.17)	0.7

*OR, odds ratio; seroprevalence includes VCA and/or EBNA-1 seropositivity*.

Secondly, SLE patients with a positive anti-EBNA-1 IgG response also form anti-EBNA348 IgG antibodies with anti-EBNA348 IgG levels being significantly higher in anti-EBNA-1 IgG positive SLE patients than in normal human controls (*p* < 0.0001) (Figure 3B).

We then investigated, whether seropositivity for EBV is also associated with antibodies against A08 of C1q and intact C1q, respectively, and whether anti-EBNA348 IgG correlate with these autoantibodies. Indeed, as shown in Figure 3C anti-A08s IgG levels were significantly higher in anti-EBNA-1 IgG positive SLE patients than in normal human controls (*p* < 0.0001). Additionally, in an inhibition assay with recombinant EBNA-1, we observed a median inhibition of anti-C1q levels by 9% (range 0–40%) in sera of SLE patients (*n* = 4 patients, *n* = 3 experiments), this being in the range of the anti-A08 specific fraction of total anti-C1q antibodies (19) (see Supplementary Figure 3).

**Figure 4:**
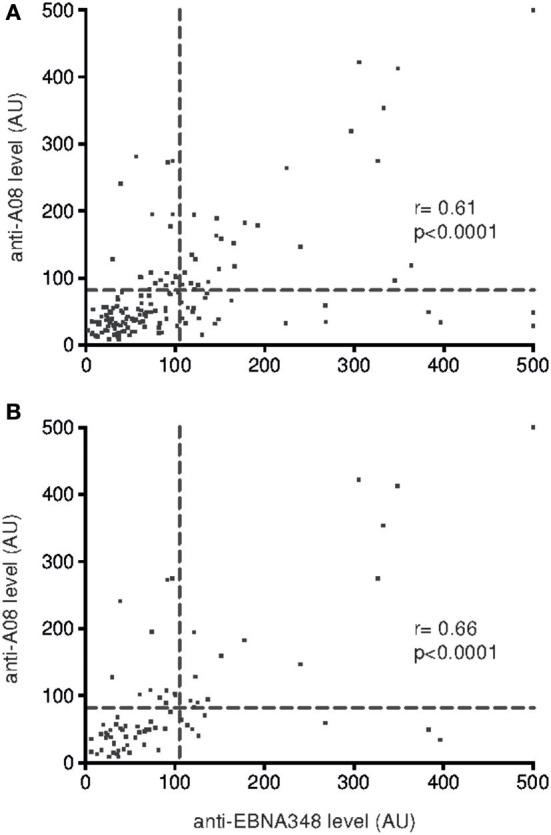
Correlation of anti-A08 IgG and anti-EBNA348 IgG serum levels in **(A)** SLE patients (CI 0.49–0.70, *n* = 165) and in **(B)** SLE patients with active disease (CI 0.51–0.78, *n* = 76). The dashed lines indicate cut-offs for anti-EBNA348 IgG (105 AU) and anti-A08 IgG (82 AU) serum levels.

Furthermore, the correlation of anti-EBNA348 IgG levels with levels of anti-A08 IgG was stronger in unselected SLE patients (*r* = 0.61, Figure 4A) than in healthy controls (*r* = 0.5) and was most pronounced in SLE patients with active disease (*r* = 0.66, Figure 4B). Double-positivity (for anti-EBNA348 IgG and anti-A08 IgG) was found only once among healthy donors, while normal human donors more frequently showed high anti-EBNA348 IgG levels without corresponding anti-A08 IgG levels (Supplementary Table 5).

Last, anti-C1q levels were significantly higher in anti-EBNA348 IgG positive SLE patients (*p* = 0.019) (Supplementary Figure 1A), and vice versa, SLE patients with positive anti-C1q had higher levels of anti-EBNA348 IgG, *p* = 0.006. This difference remains significant even after exclusion of data points with anti-C1q levels below 15 AU, i.e., exclusion of anti-C1q negative individuals (Supplementary Figure 1B). In comparison, levels of anti- ß2GPI IgG antibodies were not found to be associated with positivity for anti-EBNA348 IgG (Supplementary Figure 2).

### EBNA348 Peptide Can Induce a Humoral Immune Response to C1q *in vivo*

**Figure 5:**
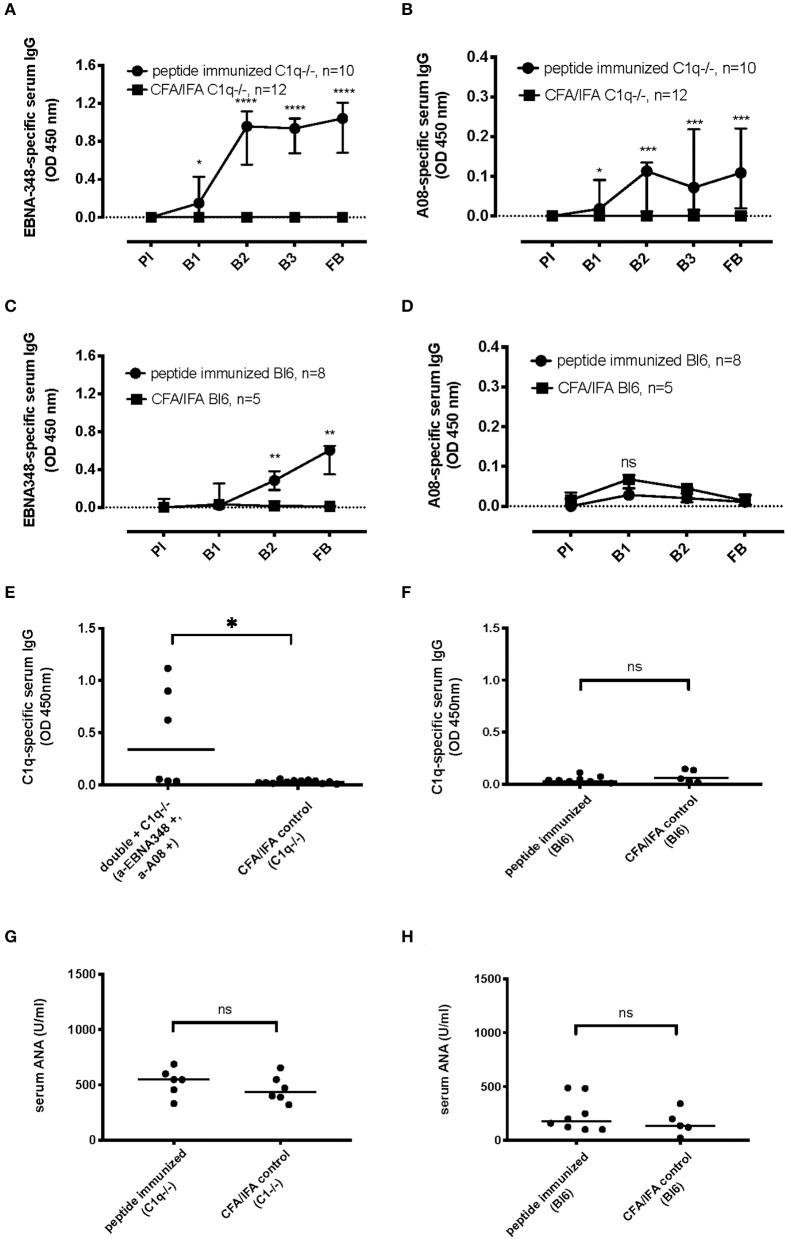
Specific humoral immune response and cross-reactivity in EBNA348 peptide-immunized C57BL/6N and C1qa^−/−^ mice. **(A)** In C1qa^−/−^ mice the EBNA348 peptide-specific immune response is associated with **(B)** measurable levels of cross-reacting anti-A08 IgG, *n* = 10. Immunized wild-type mice (C57BL/6N), have a specific anti- EBNA348 immune reaction **(C)**, but no anti-A08 IgG were detected **(D)**. **(E)** Levels of high-affinity anti-C1q antibodies in the sera (FB) of C1q^−/−^mice, positive for anti-EBNA348 IgG and anti-A08 IgG, *n* = 6, *p* = 0.01, and in the sera (FB) of C57BL/6N mice, *n* = 8 **(F)**. Animals of the control groups (*n* = 12 for C1q^−/−^ and *n* = 5 forC57BL/6N) had no IgG against any of the measured peptides or C1q. **(G,H)** Serum ANA levels in the sera (FB) of C1q^−/−^ and wild-type mice respectively, showed no significant difference between the EBNA348-immunized and the control groups. Data were plotted as median with IQR. PI, pre-immune; B1, 1st boost; B2, 2nd boost; B3, 3rd boost; FB, final bleed.

In order to confirm our patient data-based hypothesis that an EBV-derived antigenic site can induce an immune response against complement C1q, C1qa-deficient mice (*n* = 13) were immunized with the EBNA348 peptide. Of the immunized mice, 10 developed specific anti-EBNA348 antibodies (Figure 5A) and also developed an immune response against the C1q-derived A08 peptide (Figure 5B). No antibodies, either against EBNA348, or A08 could be detected in the adjuvant control group (*n* = 12). Of note, we also immunized wild-type C57BL/6N mice with the EBNA348 peptide (*n* = 10). Of the 9 mice that could be analyzed, 8 developed a peptide-specific immune response (Figure 5C), but antibodies against the A08 peptide were not detected (Figure 5D). As in C1q-deficient mice, no anti-EBNA348- or anti-A08 IgG were observed in the adjuvant control group (*n* = 5). When comparing the EBNA348-specific immune response levels between wild-type and C1q^−/−^ mice we found a significant difference right after the second boost (B2) and at the final bleeding (FB). In case of the anti-A08 IgG levels, the difference between the two strains becomes apparent at the final bleeding (see Supplementary Table 6).

We also measured anti-C1q IgG in the sera of C1q^−/−^ and wild-type mice, in immunized and control serum samples. Among the EBNA348-immunized mice showing continuous and increasing levels of anti-A08 IgG (*n* = 6), 3 had antibodies binding to intact human C1q (Figure 5E). On the other hand, none of the C57BL/6N immunized mice (*n* = 8) had anti-C1q IgG (Figure 5F). Differences in positivity for anti-C1q as well as in levels of anti-C1q were significant in immunized C1q^−/−^ mice (two tailed Mann-Whitney, *p* = 0.01, and Fisher's exact test, *p* = 0.024), whereas there was no such difference in the wild-type mice (two tailed Mann-Whitney, *p* = 0.43, and Fisher's exact test, *p* > 0.99).

Of note, we also measured ANA in the sera of C1q^−/−^ and wild-type mice (Figures 5G,H) and observed no difference between the peptide immunized and control mice (two tailed Mann-Whitney *p* = 0.41 and *p* = 0.52, respectively).

## Discussion

Although the waste disposal hypothesis provides a fundamental explanation for the development of autoantibodies, additional environmental factors, such as infectious agents, seem to be required to drive the autoimmune process.

In fact, in EBV infected SLE patients an altered virus-specific immune response against EBNA-1 epitopes can lead to antibodies cross-reacting with common lupus antigens. However, the cross-reacting antibodies described thus far neither seem to clearly correlate with disease activity nor are they commonly considered pathogenic. Our study suggests that anti-C1q in SLE patients can be induced by an EBV-derived peptide epitope through molecular mimicry. This observation is of importance since cross-reactivity as observed here seems to be based on sequence identity, and the target (C1q), as well as autoantibodies against it (i.e., anti-C1q) have been implicated in the pathogenesis of SLE (27).

Multiple lines of evidence support the involvement of C1q in SLE development. Loss of function mutations in C1q, although a rare event, are the strongest known susceptibility factor for the development of SLE (28). Additionally, active kidney inflammation (lupus nephritis) in C1q-sufficient SLE patients is not only associated with low levels of C1q but also with high levels of anti-C1q (13, 29). It is most likely that anti-C1q are altering the course of disease, especially since they have been shown to be deposited in glomeruli of patients with lupus nephritis (30), and seem to be a prerequisite for the occurrence of severe lupus nephritis (29). Concerning the binding site of anti-C1q, a linear antigenic region within the A chain of the C1q molecule (A08) was described as a major target in SLE patients (19). Now we describe that antibodies specific for the EBNA-1-derived EBNA348 peptide cross-react with A08 of C1q, which links a known risk factor for SLE with a pathogenic autoantibody in lupus nephritis.

Although we and others observed no significant difference in EBNA-1 IgG seroprevalence between SLE patients and controls (31), in SLE patients faulty and/or insufficient immune and tolerance mechanisms contribute to an aberrant anti-EBNA-1 immunity (7, 32, 33). This, on the one hand results in antibodies targeting more and different epitopes as compared to anti-EBNA-1 positive normal controls, and on the other hand can promote cross-reactive antigenic regions (32, 33).

In EBV carriers who do not suffer from SLE, the major immunogenic domain of EBNA-1 appears to be the Gly-Ala repeat region, while ligand-binding domains of EBNA-1 tend to be non-immunogenic (34). In contrast, antibodies in pediatric SLE patients preferentially target the ligand-binding N- and C-terminal parts of EBNA-1 (33). Our findings are in line with the observation, that a substantial fraction of the SLE patients (28.5%) had antibodies targeting the EBNA-1-derived region EBNA348, while these antibodies were found in <5% of the matched controls. This peptide is part of the C-terminal Gly-Arg repeat region of EBNA-1, which is functionally important for its interaction with the host chromosome (35, 36).

Previous studies have reported several different regions of EBNA-1 being recognized by antibodies specific for host proteins such as splicesomal ribonucleoproteins SmB/B', SmD, and Ro (7, 8, 33, 37, 38). James et al. were the first to show that a proline rich region of Sm B/B' antigen is very similar to a stretch of the non-structured C- terminal part of the EBNA-1 protein (AAs 397-404), and that SLE patients, but not healthy controls, have antibodies targeting this epitope (37). Anti-Sm antibodies have no direct pathogenic role or association with disease activity. However, anti-Ro antibodies are considered to be the strongest risk factor for neonatal lupus (39) and are an early marker of developing lupus, but they do not correlate with disease activity. Most importantly, anti-Ro antibodies specifically bind a peptide-defined region in the unstructured N-terminus of the EBNA-1 protein (AAs 58-72) (6). In this particular case, it is noteworthy that there is no sequence identity between the two epitopes.

In our study, we found a sequence identity of 5 consecutive AAs, namely GRRGR, between the EBNA-1-derived EBNA348 and the C1q-derived A08 stretch. The core sequence responsible for antibody binding is the RRGR. Although highly charged, it is important to mention that ionic interactions alone are not sufficient for antibody binding, as demonstrated by substitution peptide screening and an alanine scan of the A08 peptide. Nevertheless, given the length and the lack of uniqueness of the core amino acid sequence it is not inconceivable that anti-C1q/A08 can recognize other autoantigens carrying it, as suggested by the cross-reaction with the (intracellular) cell death regulator Aven (AVEN). However, sera of normal blood donors were found to also have relevant reactivity against the AVEN-derived peptide suggesting that this binding is not SLE-specific. In fact, little is known about the function of AVEN. The molecule is mainly associated with cancers, either as an anti-apoptotic molecule functioning as an oncoprotein (40) or as a predictive biomarker (41). Although the focus of our present study was on cross-reactivity with infectious agents and not on intrinsic proteins, we think that this finding warrants further investigation.

Furthermore, the binding of anti-A08 IgG was found to not only depend on the core sequence identity but also on the flanking sequences. Control peptides derived from pathogens or human molecules, with the same core region as A08, were not recognized. This might be due to charge distribution, hydrophobic residue distribution, a combination of these and /or other factors that might affect the correct epitope conformation (42–44).

In the observational part of our study one major finding was that EBNA-1 seropositive SLE patients having significantly higher anti-EBNA348 IgG levels, also had significantly higher levels of anti-A08 IgG. This finding strongly supports our data and is even more striking when considering the fact that the few normal controls having antibodies against EBNA348 did not cross-react with A08. This observation is well in line with the study by Marchini et al. (45) showing that anti-EBNA-1 antibodies only cross-react with Sm epitope in SLE patients but not in healthy controls. However, in SLE patients no clear link between anti-EBNA-1 IgG and anti-C1q could be determined. This lack of association is most likely due to different kinetics of the antibodies. While anti-EBNA-1 IgG levels seem to be more stable over time (46), anti-C1q titers strongly depend on disease activity at the time of blood sampling (47) and can even become negative in patients with inactive disease. Accordingly, we found the strongest correlation between anti-EBNA348 IgG and anti-A08 IgG in patients with active disease as determined by the Physician's Global Assessment. However, to unmask genuine cross-reactivity between anti-EBNA348 IgG and anti-A08 IgG in patients, one would have to measure antibody levels at an early stage of the disease. This perspective is based on the hypothesis that in the initial phase of the EBNA-1 immune response antibodies against EBNA348, that directly cross-react with A08 (based on sequence identity), are also present. However, anti-C1q are not only consisting of antibodies targeting A08, but are known to be polyclonal and recognize a number of different epitopes, among which A08 is still an important one but by far not the only one. A well-conceivable explanation of this phenomenon is epitope spreading eventually leading to the secondary appearance of new anti-C1q that are not cross-reacting with EBNA-1 any more but which are anti-C1q specific. Thus, cross-reacting anti-EBNA348/anti-A08/anti-C1q are likely to be an early event in the course of the disease. On the level of a classical patient with systemic lupus erythematosus having advanced disease and being years after the primary infection with EBV we would expect that the majority of anti-C1q are not of cross reacting nature any more but still contain a significant (i.e., detectable) fraction that is not only targeting A08 but also cross-reacting with EBNA-1. Vice versa, most antibodies targeting full EBNA-1 are not targeting EBNA348 (34) and thus have no obvious potential to cross-react with C1q because of a lack of sequence homology in these parts of the molecule. Based on these considerations we would expect that only a minor fraction of all anti-EBNA-1 IgG in an adult patient have the potential to cross-react with C1q. Taken together, we think that in patients with advanced SLE there is only a small fraction of cross-reacting antibodies at the protein level with the much larger fraction containing unrelated antibodies. In line with this hypothesis, we could achieve a median inhibition of 9% of anti-C1q IgG levels when incubating them with recombinant EBNA-1.

Last, we induced “*de novo* synthesis” of cross-reacting antibodies and eventually anti-C1q in animals that have no intrinsic anti-C1q. By that we confirmed our hypothesis that anti-C1q in SLE patients might be promoted by an EBNA-1-derived peptide epitope, *in vivo*. Interestingly, while immunization of C1qa^−/−^ mice with the EBNA348 peptide clearly induced antibodies cross-reacting with A08 of C1q as well as intact C1q, in C57BL/6N mice antibodies against A08 could only be detected at very low level in one out of eight mice. A well conceivable explanation for this is that the murine C1q in C57BL/6N mice induces tolerance to the GRRGR sequence-comprising epitope. For this reason we chose C1q^−/−^ mice that lack intrinsic C1q that would prevent a healthy individual from raising anti-Cq autoantibodies. This way we could observe and measure “*de-novo* synthesis” of anti-C1q as a consequence of the immunization with EBNA348, which would not have been possible in wild-type mice.

Furthermore, the fact that ANA levels at the time of the anti-C1q response show no significant difference between the peptide immunized and CFA/IFA controls, neither in wild-type nor in C1q^−/−^ mice, together with the significantly higher anti-C1q levels in the C1q^−/−^ EBNA348-immunized mice, confirm that the anti-A08 and anti-C1q response is specific.

In summary, we provide evidence that anti-C1q in SLE patients could arise from an anti-EBNA-1 immune response. The present observational data in clinical samples as well as the *in vivo* findings suggest a putative role of EBV in the development of SLE, and should be viewed as a step in understanding the mechanism of pathogen-induced autoantibodies in SLE. We speculate that, e.g., an EBV vaccination could dampen the development of cross-reactive antibodies in individuals genetically predisposed to SLE. However, EBV vaccines to prevent primary infection or therapeutic vaccines to reduce the viral load in EBV-associated malignancies have not yet been licensed. As an alternative strategy, selective immune-modulators might be able to suppress the production of cross-reacting autoantibodies in EBV-infected individuals with genetic predisposition to autoimmunity.

## Data Availability Statement

All datasets generated for this study are included in the article/Supplementary Material.

## Ethics Statement

The human study was carried out in accordance with the recommendations of the Ethical Committee of the Canton Basel, Switzerland (Ref No. EK 262/06) and Swissethics (Ethical Committee of the Canton Vaud, Switzerland Ref No. 2017-01434). All subjects gave written informed consent in accordance with the Declaration of Helsinki. The animal study was carried out in accordance with the recommendations of the Swiss welfare legislation (consisting of Animal Welfare Ordinance, Animal Welfare Act and the Animal Experimentation Ordinance). The protocol was approved by the Cantonal Commission for Animal Experiments, and the Federal Food Safety and Veterinary Office (2633/23801), and performed by authorized staff.

## Author Contributions

DV, KC, and MT conceived the study. DV, KC, LS, and ET designed and performed the experiments, analyzed and interpreted (MT) the data and performed statistical analyses. PR-L performed experiments. CR, CC, and UH-D organized and curated the SLE patient samples and data in different centers belonging to the SSCS. KC, LS, and MT wrote the manuscript. All authors participated in the critical revision of the manuscript and approved its submission for publication.

### Conflict of Interest

The authors declare that the research was conducted in the absence of any commercial or financial relationships that could be construed as a potential conflict of interest.
