# Supplementary material for: Anti-C1q Antibodies as Occurring in Systemic Lupus Erythematosus Could Be Induced by an Epstein-Barr Virus-Derived Antigenic Site
Source: Front Immunol. 2019 Nov 7;10:2619. doi: 10.3389/fimmu.2019.02619 (PMC6853867; doi:10.3389/fimmu.2019.02619)
Supplement: Supplementary file 1 [file Data_Sheet_1.docx]

**Supplementary Material**

**Supplementary Figure 1**

Figure S1. Levels of anti-C1q in sera positive for anti-EBNA-348 IgG and vice versa. (**A**) Anti-C1q levels of SLE patients positive for anti-EBNA348 IgG (●, n=47) compared to anti-EBNA348 IgG negative (▼, n=118) ones (p=0.019). (**B**) Anti-EBNA348 IgG levels of SLE patients positive for anti-C1q (●, n=88) compared to those of SLE patients negative for anti-C1q (▼, n=77) (p=0.006). Error bars indicate median serum levels and IQR.

**Supplementary Figure 2**

Legend S2: Levels of anti-β2 GPI IgG in sera positive for anti-EBNA348 IgG and vice versa. (**A**) No difference in anti-β2 GPI IgG levels of SLE patients positive for anti-EBNA348 IgG compared to anti-EBNA348 IgG negative ones was found (p=0.23). Anti-β2 GPI IgG levels (**B**) of SLE patients positive for anti-C1q were compared to those of SLE patients negative for anti-C1q. There was no difference in anti-β2 GPI IgG levels (p=0.41). Error bars indicate median serum levels and IQR.

**Supplementary Figure 3**

Legend S3: EBNA-1 molar excess lowers anti-C1q levels in SLE patient’s sera to a different extent (panels 1-4; each dot represents one technical repetition, n= 4; 6 for patient 2). Average values of anti-C1q levels before and after inhibition are summarized in the last panel. ***p* < 0.005, paired t test

**Supplementary tables**

Supplementary Table 1. Demographics of SLE patients

| ***Demographics of SLE patients*** | | | |
| --- | --- | --- | --- |
| **Sex (female in %)** | |  | |
| SLE (n= 180) | 86,2% female |  | |
| NHS (n= 189) | 85.6% female |  | |
| **Age (median, range)** | |  | |
| SLE | 43 y (16-84) |  | |
| NHS | 49 y (19-81) |  | |
| **SLE patient characteristics** | |  | |
| **SLEDAI at time of sampling (median, range)** | | 4 (0-38) | |
| **SLE disease duration in years (median, range)** | | 6 (0-52) | |
| **ACR criteria at inclusion (median, range)** | | 5 (2-11) | |
| **History of SLE nephritis (n, %)** | | n=61, (33.9%) | |
| **Ethnicity of SLE patients (n, %)** | **SLE overall** | **Active SLE** | **Inactive SLE** |
| Caucasian | 140 (77.8%) | 56 (73.7%) | 84 (80.8%) |
| African | 14 (7.8%) | 7 (9.2%) | 7 (6.7%) |
| Asian | 16 (8.9%) | 8 (10.5%) | 8 (8%) |
| Pacific Islander | 1 (0.6%) | 0 (0%) | 1 (1%) |
| Native American | 2 (1.1%) | 2 (2.6%) | 0 (0%) |
| mixed | 7 (3.9%) | 3 (4%) | 4 (3.9%) |
| **Autoantibodies in SLE (n, %)** |  |  |  |
| ANA positive | 174 (96.7%) | 73 (96.1%) | 101 (97.1%) |
| Anti-dsDNA antibodies positive | 121 (67.2%) | 50 (65.8%) | 71 (68.3%) |
| Anti-Sm antibodies positive | 40 (22.2%) | 23 (30.3%) | 17 (16.4%) |
| Anti-phospholipid antibodies positive | 86 (47.8%) | 36 (47.4%) | 50 (48.1%) |
| **Comorbidities in SLE (n, %)** |  |  |  |
| Cardio-vascular and respiratory | 27 (9.4%) | 11 (14.5%) | 16 (15.4%) |
| Metabolic-endocrine-renal | 53 (29.4%) | 21 (27.6%) | 32 (30.8%) |
| Chronic Infections | 6 (3.3%) | 2 (2.6%) | 4 (3.9%) |
| Malignancy | 13 (7.2%) | 4 (5.3%) | 9 (8.7%) |
| Associated autoimmune disorders | 50 (27.8%) | 20 (26.3%) | 30 (28.9%) |

Supplementary Table 2. Characteristics of selected SLE patients

| **patient ID** | **sex** | **age (y)** | **ACR criteria** | **LN history** | **LN status** | **anti-C1q level (AU)**^a^ | **anti-A08 level (AU)**^b^ |
| --- | --- | --- | --- | --- | --- | --- | --- |
| *SLE1* | f | 28 | 8 | yes | active | 154 | 578 |
| *SLE2* | m | 41 | 4 | yes | active | 923 | 435 |
| *SLE3* | f | 20 | 7 | yes | active | 1000 | 642 |
| *SLE4* | f | 39 | 6 | yes | active | 661 | 408 |
| *SLE5* | f | 24 | 5 | yes | active | 589 | 794 |
| *SLE6* | f | 66 | 7 | yes | active | 342 | 1480 |
| *SLE7* | f | 32 | 7 | yes | inactive | 315 | 635 |
| *SLE8* | m | 35 | 7 | yes | active | 268 | 942 |
| *SLE9* | f | 19 | 5 | yes | active | 461 | 696 |
| *SLE10* | f | 40 | 4 | yes | active | 934 | 563 |
| *SLE11* | f | 44 | 6 | yes | active | 401 | 689 |
| *SLE12* | f | 26 | 4 | yes | inactive | 432 | 822 |
| *SLE13* | f | 47 | 6 | yes | inactive | 180 | 630 |
| *SLE14* | m | 52 | 5 | no | never | 142 | 1000 |
| *SLE15* | f | 38 | ≥4 | no | never | 9 | 285 |
| *SLE16* | m | 63 | 4 | yes | inactive | 8 | 236 |
| *SLE17* | f | 58 | 5 | no | never | 24 | 259 |

Information regarding age and LN status was registered at the time of the blood sampling.

^a^ Cut-off for positivity 89 AU; ^b^ Cut-off for positivity 341 AU (the cut off is taken from an earlier and published version of the anti-A08 ELISA). LN: lupus nephritis

Supplementary Table 3. Selected protein-derived peptides with the RRGR (core) sequence

| **Micro-organism related protein-derived peptides** | |
| --- | --- |
| *Lactobacillus johnsonii* | IDNGRRGRPVVGP |
| *Streptococcus pyogenes* | IDNGRRGRPITGP |
| *Mycobacterium tuberculosis* | FDNGRRGRPVTGP |
| Epstein-Barr virus: EBNA-1 _348-364_ | GSGGRRGRGRERARGGS |
| **Human protein-derived peptides** | |
| Cell death regulator Aven-derived peptide _46-58_ | DGGGRRGRGRGRG |
| Collagen type XI-alpha-2 _532-544_ | GKAGRRGRAGADG |
| Semaphorin-4G _701-713_ | LREGRRGRRRKYS |
| Phosphofurin acidic cluster sorting protein-2 _427-439_ (PACS-2) | KQAGRRGRSTSLK |
| Collagen type V-alpha-1 _604-616_ | GKPGRRGRAGSDG |
| AChE Q subunit _126-138_ | GRPGRKGRPGPPG |
| scr A08 | RPRGLRGPRGGKG |

Subscript numbers indicate amino acid positions in protein. Colored residues indicate sequence identity with A08 peptide: GRPGRRGRPGLKG

Supplementary Table 4. C1q-derived A08 sequence alignment

| Species | | | | | | | | | | | | | |
| --- | --- | --- | --- | --- | --- | --- | --- | --- | --- | --- | --- | --- | --- |
| HUMAN | **G_1_** | **R_2_** | **P_3_** | **G_4_** | **R_5_** | **R_6_** | **G_7_** | **R_8_** | **P_9_** | **G_10_** | **L_11_** | **K_12_** | **G_13_** |
| *Non-Human Primates* |  |  |  |  |  |  |  |  |  |  |  |  |  |
| Chimpanzee (great ape) | G | R | P | G | R | P | G | R | P | G | L | K | G |
| Rhesus macaque(OWM) | G | R | P | G | R | P | G | R | P | G | L | K | G |
| Marmoset (NWM) | G | R | P | G | R | P | G | R | P | G | L | K | G |
| *Rodents* |  |  |  |  |  |  |  |  |  |  |  |  |  |
| Mouse | G | N | P | G | R | P | G | R | P | G | L | K | G |
| Rat | G | I | P | G | R | P | G | R | P | G | L | K | G |
| Guinea Pig | G | K | P | G | R | P | G | R | P | G | L | K | G |
| Hamster | G | N | P | G | R | P | G | R | P | G | P | K | G |
|  |  |  |  |  |  |  |  |  |  |  |  |  |  |
| Rabbit | G | K | P | G | R | P | G | R | P | G | L | K | G |
| Sheep | G | I | P | G | R | P | G | R | P | G | L | K | G |
| Goat | G | I | P | G | R | P | G | R | P | G | L | K | G |
| Cattle | G | I | P | G | R | P | G | R | P | G | L | K | G |
| Pig | R | K | P | G | R | P | G | R | P | G | P | K | G |
| Horse | G | I | P | G | R | P | G | R | P | G | P | K | G |
| Donkey | G | I | P | G | R | P | G | R | P | G | P | K | G |
| Dog | G | T | P | G | R | P | G | R | P | G | L | K | G |
| Zebrafish | G | P | N | G | R | D | G | L | P | G | P | K | G |

Subscript numbers indicate amino acid positions in the A08 peptide. The corresponding residues in the A-chain of human C1q are AA 37 – 49. OWM: Old World monkeys, NWM: New World monkeys. Colored letters indicate deviation from the human sequence.

Supplementary Table 5. Distribution of anti-EBNA348 and anti-A08 in SLE and NHS

| **SLE** | n=165 (EBNA1+, VCA+) | | **NHS** | n=171 (EBNA1+, VCA+) | |
| --- | --- | --- | --- | --- | --- |
|  | **EBNA348 +** | **EBNA348 -** |  | **EBNA348 +** | **EBNA348 -** |
| **A08 +** | 27 (16.36%) | 20 (12.12%) | **A08 +** | 1 (0.58%) | 6 (3.51%) |
| **A08 -** | 20 (12.12%) | 98 (59.39%) | **A08 -** | 7 (4.09%) | 157 (91.81) |
| **sum** | 47 (28.48%) | 118(71.52%) | **sum** | 8 (4.68%) | 163 (95.32%) |

Supplementary Table 6. Comparison of peptide-specific immune response in C57BL/6N and C1q^-/-^ mice

| **Timepoint** | **anti-EBNA348** | **anti-A08** |
| --- | --- | --- |
| B1 | ns (0.99) | ns (0.62) |
| B2 | *** (0.0005) | ns (0.097 |
| FB | * (0.015) | * (0.0314) |

Mann-Whitney test on n= 8 BL6 mice and n=10 C1q^-/-^
